# Supplementary material for: The causal relationship between the human gut microbiota and pyogenic arthritis: a Mendelian randomization study
Source: Front Cell Infect Microbiol. 2024 Nov 26;14:1452480. doi: 10.3389/fcimb.2024.1452480 (PMC11629706; doi:10.3389/fcimb.2024.1452480)
Supplement: Supplementary file 1 [file DataSheet1.zip › mendelian test/ebi-a-GCST90027562.csv_forest.pdf]

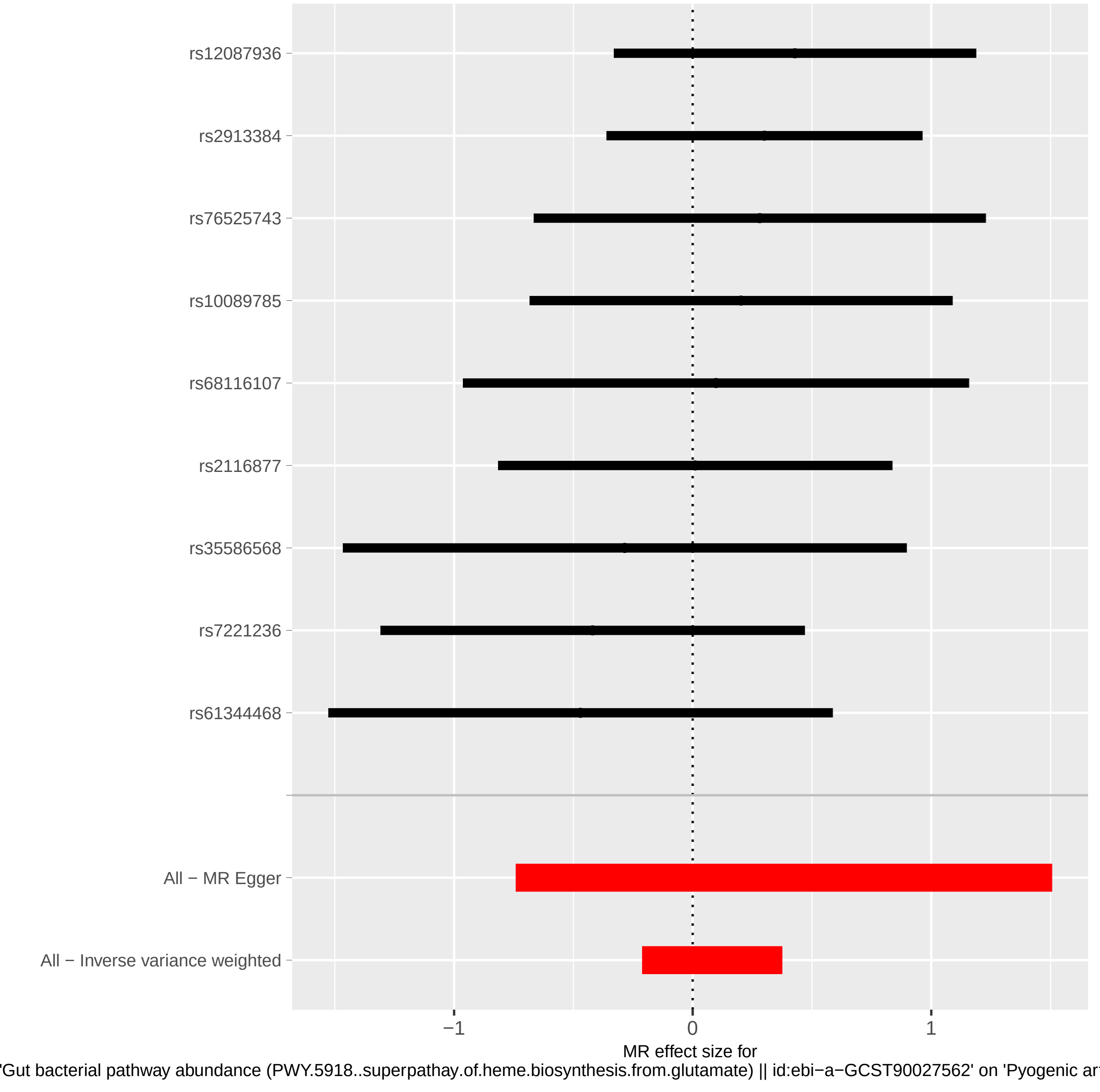
rs12087936
rs2913384
rs76525743
rs10089785
rs68116107
rs2116877
rs35586568
rs7221236
rs61344468
All − MR Egger
All − Inverse variance weighted
−1
0
1
MR effect size for
'Gut bacterial pathway abundance (PWY.5918..superpathay.of.heme.biosynthesis.from.glutamate) || id:ebi−a−GCST90027562' on 'Pyogenic ar
